# Bispecific antibody-targeted T-cell therapy for acute myeloid leukemia

**DOI:** 10.3389/fimmu.2022.899468

**Published:** 2022-11-01

**Authors:** Ewa Kubicka, Lawrence G. Lum, Manley Huang, Archana Thakur

**Affiliations:** Cellular Immunotherapy and Bone Marrow Transplant Program, Department of Medicine, Division of Hematology/Oncology, University of Virginia, Charlottesville, VA, United States

**Keywords:** targeted T cells, bispecific antibodies, acute myeloid leukemia, anti-CD123, anti-CD33

## Abstract

The management of relapsed or refractory acute myeloid leukemia (AML) continues to be therapeutically challenging. Non-toxic immunotherapy approaches are needed to provide long-term anti-leukemic effects. The goal of this study was to determine whether activated T cells (ATCs) armed with bispecific antibodies (BiAbs) could target and lyse leukemic and leukemic stem cells (LSCs). Anti-CD3 × anti-CD123 BiAb (CD123Bi) and anti-CD3 × anti-CD33GO (gemtuzumab ozogamicin [GO]) BiAb (CD33GOBi) were used to arm ATCs to produce bispecific antibody armed activated T cells (designated CD123 BATs or CD33GO BATs) to target AML cell lines, peripheral blood mononuclear cells from AML patients, and *in vivo* treatment of AML in xenogeneic NSG mice engrafted with leukemic cells. BATs exhibited high levels of specific cytotoxicity directed at AML cell lines at low 1:1 or 1:2 effector-to-target (E:T) ratios and secrete Th_1_ cytokines upon target engagement. *In vivo* study in AML-engrafted NSG mice showed significantly prolonged survival in mice treated with CD33GO BATs (p < 0.0001) or CD123 BATs (p < 0.0089) compared to ATC-treated control mice. Patient samples containing leukemic blasts and LSCs when treated with CD33GO BATs or CD123 BATs for 18 h showed a significant reduction (50%–100%; p < 0.005) in blasts and 75%–100% reduction in LSCs (p < 0.005) in most cases compared to unarmed ATCs. This approach may provide a potent and non-toxic strategy to target AML blasts and LSCs and enhance chemo-responsiveness in older patients who are likely to develop recurrent diseases.

## Introduction

Acute myeloid leukemia (AML) is a heterogeneous disease characterized by the accumulation of malignant cells in the myeloid lineage that poses a significant therapeutic challenge (1). Although allogeneic stem cell transplant (AlloSCT) provides a long-term cure for high-risk patients who have an AlloSCT donor, there are significant regimen-related toxicities and a high risk of relapse (2–8). Recurrent disease is attributed to leukemic stem cells (LSCs), which are thought to be resistant to chemotherapy and capable of reinitiating the disease. Leukemic antigens CD33 and CD123 are highly expressed on blasts and LSCs, making them attractive AML targets (9, 10). Although a humanized anti-CD33 IgG4 antibody conjugated to the cytotoxic agent calicheamicin (gemtuzumab ozogamicin [GO]) was reapproved for treating newly diagnosed AML in relapsed/refractory (R/R) AML in adults and pediatric patients, treatment with GO continues to be associated with serious adverse effects (11, 12). Several bispecific antibodies, trispecific antibodies, drug conjugates, and CAR T cell-based studies to target CD33 or CD123 are ongoing but have not reproduced the efficacy seen in acute lymphocytic leukemia (ALL) (13–16). Thus, novel non-hematotoxic therapeutic strategies that target and eradicate LSCs and AML blasts are urgently needed. Our bispecific antibody armed activated T-cell (BAT) approach using anti-CD3 × anti-CD33 BiAb (CD33GOBi) redirected T cells (CD33GO BATs) or anti-CD3 × anti-CD123 BiAb (CD123Bi) redirected T cells (CD123 BATs) may improve outcomes for patients with AML without cytokine release syndrome (CRS) and may enhance chemo-responsiveness of chemo-resistant cells in a non-MHC restricted manner.

In our preclinical studies, we have shown that engagement between target and effector cells created by the anti-CD3 × anti-tumor associated antigen (TAA) BiAb triggers the non-MHC restricted release of perforin/granzyme B that kills tumor targets, increasing T-cell trafficking and secretion of Th_1_ cytokines (17, 18). In BAT-based therapeutic strategy in phase I/II clinical trials, we have demonstrated the lack of CRS in solid tumors (19–23), non-Hodgkin’s lymphoma (NHL) (24), and multiple myeloma (MM) (25), combined with controlled potency by adjusting the arming dose of the bispecific antibody, the cell dose (BATs) per infusion, and the number and frequency of infusions. In addition, multiple non-toxic infusions of BATs can induce the recruitment of endogenous adaptive and innate immune cells through the non-toxic release of cytokines and chemokines to provide an improved anti-leukemia effect. BAT approach can be a versatile and promising alternative to target AML.

Our clinical studies using the same strategy as mentioned above show that HER2 BATs against breast and prostate cancers (19–22, 26), EGFR BATs against pancreatic cancer (23), and CD20 BATs against lymphoma (24) and multiple myeloma precursor cells (25) showed clinical and immunological responses without CRS. In this study, we show that ATCs armed with CD123Bi (CD123 BATs) or CD33GOBi (CD33GO BATs) exhibit high levels of specific cytotoxicity directed at CD123- and CD33-expressing cells, release Th_1_ cytokines/chemokines, and sensitize drug-resistant AML cells for chemo-responsiveness.

## Materials and methods

### Cell lines

TF1, NoMo1, EOL1, KG1, HL60, and vincristine-resistant (VCR) HL60/VCR cell lines were from Dr. Thomas P. Loughran, Jr., University of Virginia Cancer Center. The cell lines were grown in RPMI-1640 (or IMDM for KG1) supplemented with either 10% (TF1, NoMo1, HL60, and HL60/VCR) or 20% (KG1 and EOL1) fetal calf serum (FCS), 1% l-glutamine, and 2% penicillin-streptomycin except for TF1 culture media that were supplemented with additional 2 ng/ml of granulocyte-macrophage colony-stimulating factor (GM-CSF). HL60/VCR line was maintained in 1 μg/ml of vincristine.

### Activation and expansion of T cells

Research protocols for blood collection from healthy donors were approved by the University of Virginia Institutional Review Board (IRB Approved Protocol #18904). Informed consent was obtained from all normal donors. Peripheral blood mononuclear cells (PBMCs) were isolated from heparinized whole blood of normal healthy donors by Ficoll-Hypaque density gradient centrifugation and re-suspended in RPMI-1640 supplemented with 10% FCS, 1% l-glutamine (Lonza, Singapore), and 2% penicillin-streptomycin; T cells were activated with 20 ng of OKT3/ml and expanded in 100 IU of IL-2/million cells.

### Production of anti-CD3 × anti-CD123 and anti-CD3 × anti-CD33GO bispecific antibodies

OKT3, a murine anti-CD3 epsilon IgG2a monoclonal antibody (mAb), was purchased from Miltenyi Biotech (Auburn, CA, USA) and Bio X Cell (Lebanon, NH, USA). Anti-CD123 (Clone 7G3) was purchased from BD Biosciences (Franklin Lakes, NJ, USA). GO (Mylotarg™), a recombinant humanized anti-CD33 monoclonal antibody (IgG4 κ antibody hP67.6) covalently linked to the calicheamicin (anti-CD33GO), was purchased from the UVA Pharmacy (Charlottesville, VA, USA). OKT3 was chemically heteroconjugated with anti-CD123 mAb and anti-CD33GO as described (27). In brief, OKT3 was crosslinked using a 10-fold molar excess of Traut’s reagent, and anti-CD123 mAb or gemtuzumab was crosslinked using a fourfold molar excess of sulfo-SMCC. The BiAb was produced by combining crosslinked mAbs at a 1:1 ratio by overnight heteroconjugation at 4°C to produce anti-CD3 × anti-CD123 BiAb (CD123Bi) and anti-CD3 × anti-CD33GO BiAb (CD33GOBi).

### Arming of activated T cells with bispecific antibodies

CD123Bi and CD33GOBi were titrated for the optimal dose to arm activated T cells (ATCs). ATCs were armed with increasing doses of CD123Bi or CD33GOBi (0.5 to 500 ng/10^6^ ATCs) for 30 min at room temperature and were washed thrice to eliminate any unbound BiAb.

### Primary patient acute myeloid leukemia cells

AML cells from pretreatment diagnostic peripheral blood or bone marrow specimens were obtained from the Orien Biorepository at the University of Virginia Cancer Research Center (UVA Orien IRB HSR 18445) from adult patients with AML. Patients provided written informed consent for the collection and use of their biospecimens for research purposes under a protocol approved by the UVA Institutional Review Board. Clinical data were de-identified for all patients.

### Immunostaining for acute myeloid leukemia blasts and acute myeloid leukemia leukemic stem cells

PBMCs from patients diagnosed with AML were stained with anti-CD34, anti-CD 38, anti-CD90, anti-CD33, anti-CD123, and anti-TIM3 monoclonal antibodies to quantitate the expression of CD33 and CD123 on AML blasts (CD45^dim^/CD34^+/−^/CD38^+/−^), LSCs (CD34^+^/CD38^−^/CD90^−^/TIM3^+^), and hematopoietic stem cells (HSCs) (CD34^+^CD38^−^/CD90^+^/TIM3^−^) and the proportion of these cells in the PBMCs by flow cytometry. The phenotypes of these cells are also listed in **Table S1**.

### Flow cytometry-based cytotoxicity assay

We recently developed a quantitative flow cytometry-based cytotoxicity assay that is highly sensitive at low effector-to-target (E:T) ratios in which the concentration of both effector T cells (BATs) and target cells (AML cell lines) is measured in fixed-volume aliquots at the time of initiation and after 18 h (or more) of culture using an ACEA Biosciences (San Diego, CA, USA) NovoCyte flow cytometer (28). Briefly, the target cells are fluorescently labeled with eFluor 450 dye (Invitrogen, Carlsbad, CA, USA) and added to 24- or 96-well culture plates. ATCs were added to target cells at the designated E:T ratios and gently mixed. At designated time points, fixed volumes of co-cultured cells were acquired to determine the number of live BATs and target cells as previously described (28). Briefly, the tracking dye eFluor 450-positive and 7AAD-negative PBMCs represent the live AML populations after the targeted overnight killing by BATs. A forward/side scatter gate was drawn to capture the lymphocyte population followed by an enumeration of live BATs (7AAD^−^/eFluor 450^−^) and live target cells (7AAD^−^/eFluor 450^+^). The percent killing was calculated using the following formula: [1 − (number of targets cultured with effectors/number of targets cultured without effectors)] × 100.

### Flow-based cytotoxicity assay to target patient-derived leukemic stem cells

We used a quantitative flow cytometry-based cytotoxicity assay as described above. Briefly, PBMCs collected from patients at the time of diagnosis were stained with eFluor 450 dye and co-cultured with CD123 BATs, CD33GO BATs, or unarmed ATCs. Fixed volume from the co-culture at 0 h and after 18 h was stained for HSCs, AML blasts, AML LSCs, and myeloid cells followed by adding 7-AAD for quantitative measurement of cytotoxicity in the percentage of target lyse as well as the absolute reduction in the numbers of blasts or LSCs eliminated in the assays.

### Acute myeloid leukemia engraftment and treatment of mice

All animal protocols were reviewed and approved by the Institute of Animal Care and Use Committee of the University of Virginia. We used an equal ratio of male and female, 6–8-week-old NOD *scid* gamma ([NSG] NOD.Cg-*Prkdc^scid^ Il2rg^tm1Wjl^*/SzJ) mice from Jackson Laboratory; these mice have a deficiency in innate immunity and deficiency in IL-2 receptor gamma chain that disables cytokine signaling and lack mature T cells, B cells, and functional NK cells. Briefly, NSG mice were irradiated with 250 rad; after 24 h of irradiation, mice were intravenously injected with 10 × 10^6^ KG1 cells to establish human AML engraftment and were given sulfamethoxazole:trimethoprim for 1 week post irradiation. The AML engraftment was monitored by staining for human CD45^+^ cells in the flushed bone marrow (BM) on days 7, 14, and 21. Treatment was begun once engraftment reached ~15% (**Figure S4**). Mice were treated with 20 × 10^6^ cells/injection, 3×/week for 4 weeks (n = 10 mice/group). Control and treatment groups (G) include unarmed ATCs (G1), CD33GO BATs (G2), CD123 BATs (G3), and 0.06 mg/kg of Mylotarg (GO) alone (G4). Following 4 weeks of treatment, mice were monitored for survival.

We repeated the *in vivo* experiment again in the NSG mouse model using AML engraftment with KG1a cells. Treatment was started on day 21 when engraftment of CD45^+^ KG1a cells reached 30% in the bone marrow. Mice were treated with control ATCs or CD123 BATs (20 × 10^6^ cells/injection) 3×/week for 4 weeks. The end point was survival following 4 weeks of treatment.

The methods for the dose–response curve of GO to determine IC_50_ dose for AML cell lines, flow cytometry-based drug efflux assay, and cytokine/chemokine profiling are provided in the **Supplementary Material**.

### Statistical considerations

Descriptive statistics were used to analyze the data using GraphPad Prism Software Inc. (San Diego, CA, USA). The mean and standard deviation are reported. All p-values are two-tailed. All comparisons were performed using the Mann–Whitney or Wilcoxon signed-rank test.

## Results

### Production of anti-CD3 × anti-CD123 and anti-CD3 × anti-CD33 bispecific antibodies

**Figure 1:**
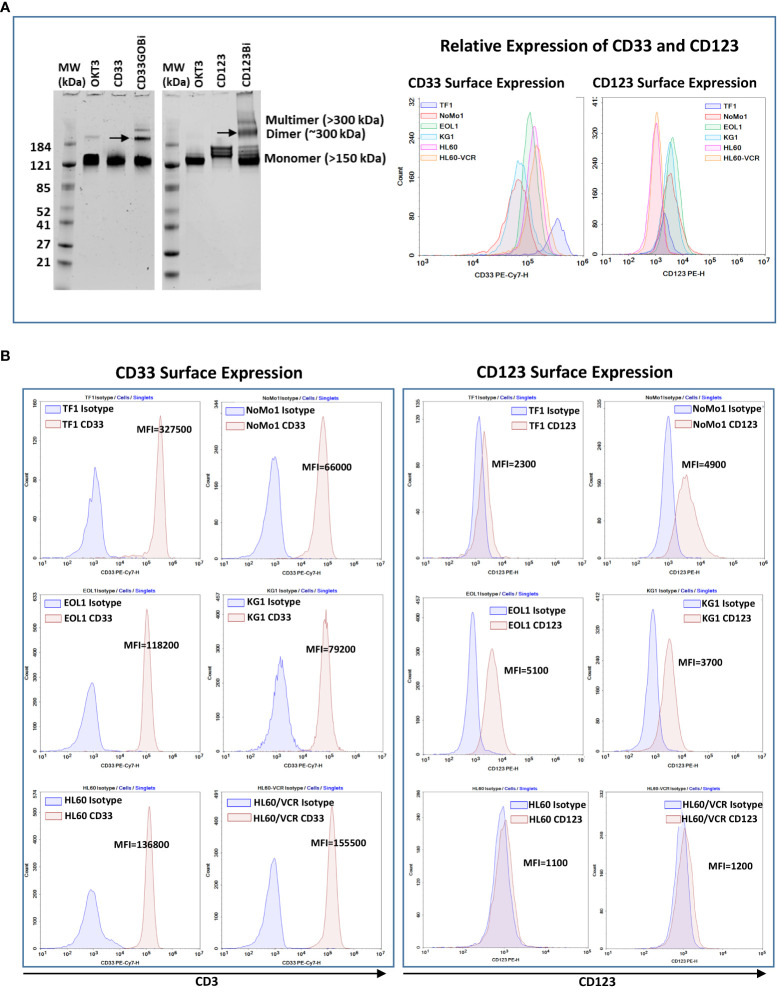
**(A)** Left panel showing SDS-PAGE images of heteroconjugated products of CD33GOBi and CD123Bi. Lane 1 in both gels: molecular weight markers (MW); Lane 2 in both gels: OKT3; Lane 3: CD33 in gel #1or CD123 in gel #2; Lane 4: CD33GOBi in gel #1 or CD123Bi in gel #2 containing unconjugated monomers, dimers (arrow), and multimers forms. Right panel shows the relative expression of CD33 and CD123 on AML cell lines. **(B)** Left panel shows surface expression of CD33 in TF1, NoMo1, EOL1, KG1, HL60 and vincristine resistant HL60 (HL60/VCR) AML cell lines. Right peak on each plot shows binding of anti-CD33 relative to isotype control mouse IgG1 (left peak). Right panel shows staining of anti-CD123 (right peak) relative to isotype control mouse IgG1 (left peak) in all six AML cell lines.

Anti-CD33GO and anti-CD123 monoclonal antibodies were heteroconjugated with OKT3 (anti-CD3) to produce CD33GOBi and CD123Bi as described (27). The BiAbs were separated on a native gel to quantitate the proportions of dimers, multimers, and unconjugated monomers. CD33GOBi showed ~6%, 19%, and 75% and CD123Bi showed ~10%, 20%, and 70% of multimers, dimers, and monomers, respectively (**Figure 1A**, left panel). Any unbound monomers and multimers are rinsed from the BAT product before performing any *in vivo* and *in vitro* experiments.

### CD33 and CD123 expression and proportion of blasts in acute myeloid leukemia cell lines

All AML cell lines (TF1, NoMo1, EOL1, KG1, HL60, and HL60/VCR) were stained for CD123 or CD33 expression using the fluorochrome-conjugated anti-CD123 and anti-CD33 antibodies. The relative expression of CD33 and CD123 is shown in the right panel of **Figure 1A**. Variable mean fluorescence intensity (MFI) of CD33 and CD123 expression on AML cell lines is shown in **Figure 1B** (left and right panels) and in **Table 1**. Co-expression of CD33 and CD123 and proportions of blasts in cell lines were detected by phenotyping for CD123, CD33, CD34, and CD38 markers. Co-expression of CD33^+^/CD123^+^ ranged from 0.58% to 78.9% (TF1, 27.8%; NoMo1, 65.7%; EOL1, 78.9%; KG1, 58.8%; HL60, 1.16%; and HL60/VCR, 0.58%). The proportions of CD33^+^/CD123^+/−^, CD34^+/−^/CD38^+/−^, and CD34^−^/CD38^+^ populations are shown in **Figures S1A, B**.

**Table 1:** Expression of CD33^+^/CD123^+^ in cell line and cytotoxicity mediated by CD33GO or CD123 BATs.

Cell lines	CD33^+^/CD123^+^ % positive cells			Expression of CD33^+^/CD123^+^ (MFI)		S/R	% Cytotoxicity (E:T 1:2)		
	CD33^+^	CD123^+^	CD33^+^/CD123^+^	CD33^+^	CD123^+^	GOS or R	CD33GO BATs	CD123 BATs	33GO/123 BATs
**TF1**	72.14	0.05	27.86	327,500	2,300	R	25	24	21
**NoMo1**	34.32	0.09	65.68	66,100	4,900	S	33	29	44
**EOL1**	21.08	0.21	78.92	118,200	5,100	S	61	56	54
**KG1**	41.14	0.01	58.86	79,200	3,700	R	41	34	40
**HL60**	98.58	0.0	1.16	136,800	1,100	S	57	50	64
**HL60/VCR**	99.27	0.01	0.58	155,500	1,200	R	51	48	66

GO, gemtuzumab ozogamicin; BATs, bispecific antibody armed activated T-cell; S, GO-sensitive cell lines; R, GO-resistant cell lines; MFI, mean fluorescence intensity.

### CD33-expressing acute myeloid leukemia cell lines show resistance to gemtuzumab ozogamicin

**Figure 2:**
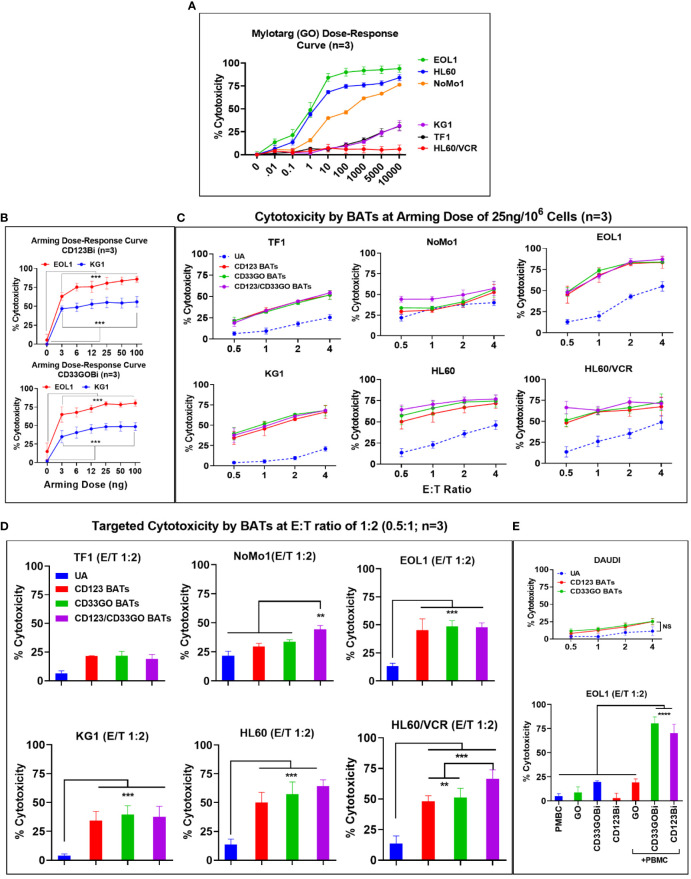
**(A)** Mylotarg Dose-Response Curve. Shows the 50% inhibitory concentration (IC50) of GO (mylotarg) in TF1, NoMo1, EOL1, KG1, HL60 and HL60/VCR cell lines. **(B)** BiAb Arming Dose-Response Curve. Normal donor ATC (n=3) were either left unarmed (UA, 0 ng/106 ATC) or armed at 3, 6, 12, 25, 50 and 100 ng/106 ATC with CD123Bi (upper panel) and CD33GOBi (lower panel) and tested against EOL1 and KG1 at E:T 1:1 in an 18 hr cytotoxicity assay. The difference was highly significant (*** = p<0.0001) at all 3, 6, 12, 25, 50 and 100 ng/106 arming doses for armed ATC compared to unarmed ATC for both cell lines using Wilcoxon signed-rank test. **(C)** Titration of E:T Ratio at BiAb Arming Dose of 25 ng/106 ATC. Cytotoxicity of CD33GO- or CD123 BATs against TF1, NoMo1, EOL1, KG1, HL60 and HL60/VCR cell lines was measured at fixed arming dose of 25 ng/106 ATC at 0.5:1, 1:1, 2:1 and 4:1 E/T ratios (n=3 normal donor ATC). Unarmed ATC (UA) or ATC armed at 25 ng/106 cells with CD123Bi, CD33GOBi or combining both BATs CD123-/CD33GO BATs at 1:1 ratio were incubated for 18 hr with AML cell lines listed above showed increasing cytotoxicity from 0.5:1 to 2:1 ratio plateauing at E:T of 4:1 for EOL1, KG1, HL60 and HL60/VCR cell lines with the exception of TF1 which showed gradual increase at E:Ts from 0.5:1 to 4:1, NoMo1 showed slightly increased cytotoxicity at 4:1 E:T ratio. Statistical analysis is presented for the lowest E/T of 0.5:1 (1 effector cell:2 target cells) in section D below. **(D)** Targeted Cytotoxicity by BATs at 0.5:1 (1:2) E/T Ratio. Unarmed ATC or ATC were armed with CD123Bi, CD33GOBi or both BATs (CD123-/CD33GO BATs) combined were tested for cytotoxicity against all six AML cell lines at 1:2 E/T ratio (n=3). Cytotoxicity was significantly higher by CD123-, CD33GO- or combined CD123-/CD33GO BATs against EOL1 (P<0.0003), KG1 (P<0.0002), HL60 (P<0.0001) and HL60/VCR (P<0.0003) compared to unarmed ATC. No significant difference in cytotoxicity of NoMo1 cells was observed when targeted with CD123- or CD33GO-, but combining CD123-/CD33GO BATs showed significantly higher cytotoxicity (P<0.008) against NoMo1 compared to ATC, CD123-, CD33GO BATs using Wilcoxon signed-rank test (** = p<0.005; *** = p<0.0005). **(E)** Upper panel shows the specificity of CD123- and CD33GO BATs, there is very low killing of CD20+/CD123-/CD33- cell line DAUDI (highest killing was ~20% at highest E/T 4:1). Statistical analysis using Wilcoxon signed-rank test between unarmed (UA) and armed cells showed no significant difference. Lower panel shows killing by PBMC alone, GO alone, CD33GOBi alone, CD123Bi alone or PBMC+GO, PBMC+CD33GOBi, PBMC+CD123GOBi. Cytotoxicity was significantly high (n=3; P<0.00001) by PBMC+CD33GOBi and PBMC+CD123Bi compared to PBMC alone, GO alone, CD33GOBi alone, CD123Bi alone or PBMC+GO at E/T 2:1 against EOL1 cell line using Wilcoxon signed-rank test (**** = p<0.00005).

AML cell lines EOL1, HL60, and NoMo1 showed 50% cytotoxicity at 1.8, 4.0, and 150 ng/ml doses of GO at 72-h cytotoxicity assay, respectively. Interestingly, the IC_50_ dose of GO for KG1, TF1, and HL60/VCR could not be reached even up to 10,000 ng/ml (10 μg/ml) concentration of GO in spite of the high expression of CD33 on these cell lines (**Figure 2A**).

### Arming dose titrations of CD33GOBi and CD123Bi

Arming dose titrations were performed at 0 (unarmed ATCs [UAs]), 3, 6, 12, 25, 50, and 100 ng of CD33GOBi or CD123Bi/10^6^ ATCs (**Figure 2B**). Mean specific cytotoxicity against EOL1 and KG1 cells plateaued respectively at 80% and 57% for CD123 BATs and 78% and 50% for CD33GO BATs at an E:T of 1:1 (**Figure 2B**, top and bottom panels). The plateau was achieved between arming doses of 25 and 100 ng/10^6^ ATCs at an E:T of 1:1; based on these data, an arming dose of 25 ng/10^6^ ATCs was used in all subsequent experiments.

### CD33GO and CD123 bispecific antibody armed activated T cells exhibit specific cytotoxicity against acute myeloid leukemia targets

Both CD33GO and CD123 BATs mediated effective and specific cytotoxicity for all AML cell lines with variable CD33 and CD123 expression at E:T ratios ranging from 0.5:1 to 4:1 except NoMo1. There was no correlation found between the proportion of CD33- or CD123-positive cells, MFI levels, and the levels of specific cytotoxicity of the different cell lines (**Table 1**).

### CD123 bispecific antibody armed activated T cells

ATCs from three normal donors armed with CD123Bi at 25 ng/10^6^ cells exhibited specific cytotoxicity at E:T ratios ranging from 0.5:1 to 4:1. The specific cytotoxicity mediated by CD123 BATs against TF1 ranged from 24% to 50%, NoMo1 from 29% to 53%, EOL1 from 56% to 83%, KG1 from 34% to 68%, HL60 from 50% to 72%, and HL60/VCR from 48% to 67% (**Figure 2C**). There was no difference in the cytotoxic activity against TF1 or NoMo1 by CD123 BATs compared to UAs, but cytotoxicity was significantly higher against EOL1 (p < 0.0003), KG1 (p < 0.0002), HL60 (p < 0.0001), and HL60/VCR (p < 0.0003) by CD123 BATs compared to UAs (**Figure 2D**).

### CD33GO bispecific antibody armed activated T cells

The specific cytotoxicity by CD33GO BATs against TF1 ranged from 25% to 49%, NoMo1 from 34% to 56%, EOL1 from 61% to 82%, KG1 from 41% to 70%; HL60 from 57% to 75%, and HL60/VCR from 51% to 73% compared to UAs which at the same E:T ratios exhibited 5%–55% cytotoxicity (**Figure 2C**). The statistical analysis at 0.5:1 E/T ratio showed a significant difference in specific cytotoxicity only for EOL1 (p < 0.0003), KG1 (p < 0.0002), HL60 (p < 0.0001), and HL60/VCR (p < 0.0003) compared to UAs at the same E:T ratio (**Figure 2D**).

### CD33GO/CD123 bispecific antibody armed activated T cells

Next, we asked whether mixing CD33GO BATs and CD123 BATs in equal amounts would exhibit enhanced specific cytotoxicity compared to the CD33GO BATs or CD123 BATs alone. Combined targeting with CD33GO BATs or CD123 BATs shows significantly enhanced cytotoxicity against NoMo1 only at a low E:T of 0.5:1 (p < 0.008), while there was no added cytotoxic effect of combined targeting against TF1, KG1, EOL1, HL60, and HL60/VCR cell lines (**Figure 2D**). At higher E:T ratios of 4:1, specific cytotoxicity against all cell lines tested showed a similar pattern as with single antigen targeting by CD33GO BATs or CD123 BATs (**Figure 2C**, six panels).

To confirm the specificity of CD123 BATs or CD33GO BATs killing AML cells, we used DAUDI cells, which do not express CD33 or CD123, at different E:T ratios of 0.5:1, 1:1, 2:1, and 4:1. Both CD33GO BATs and CD123 BATs show low levels of lymphokine-activated killer (LAK) cell-like cytotoxicity as seen by UAs against DAUDI cells (**Figure 2E**, top panel), confirming the specificity of CD33GO or CD123 BATs for AML cells. No statistically significant difference in cytotoxicity was observed between CD33GO BATs and UAs or between CD123 BATs and UAs against the DAUDI cell line.

### Low levels of cytotoxicity mediated by gemtuzumab ozogamicin alone, CD33GOBi, or CD123Bi alone

Since the IC_50_ dose of GO is 1.8 ng/ml for the EOL1 cell line, we used the same concentration for the overnight (18-h) cytotoxicity assay. Similarly, an effective dose of 5 ng BiAb/well of CD33GOBi or CD123Bi against the EOL1 cell line was used. Since the mechanism of action of GO is by internalization and cleavage to release calicheamicin independent of antibody-dependent cellular cytotoxicity (ADCC), we carried out cytotoxicity assays for 18 h to allow internalization of GO. Cytotoxicity with GO alone, CD33GOBi, or CD123Bi alone was 8.7%, 19.5%, and 2.9%, respectively, in the absence of PBMCs. In the presence of PBMCs (E:T ratio, 2:1), both CD33GOBi and CD123Bi showed significantly higher cytotoxicity of 80% (p < 0.00001) and 70% (p < 0.00001) respectively, compared to PBMCs alone, GO alone, and BiAbs alone (**Figure 2E**, bottom panel).

### Cytokine release profile in targeting killing of acute myeloid leukemia cells by bispecific antibody armed activated T cells

**Figure 3:**
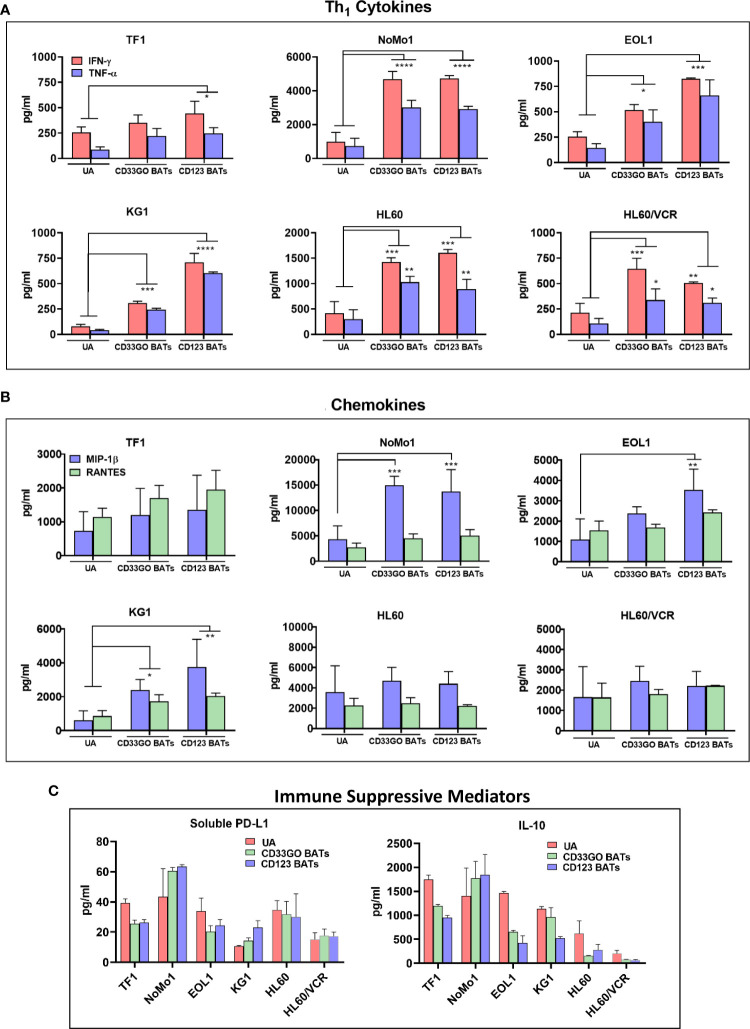
Cytokines/Chemokines Production upon BATs Engagement of AML cells. Cell-free supernatants were collected after 18 hr co-cultures of TF1, NoMo1, EOL1, KG1, HL60 and HL60/VCR cell lines with unarmed ATC, CD33GO- or CD123 BATs at E:T of 10:1 (n=3) for cytokine/chemokine profiling using 3 normal donor ATC or BATs. **(A)** Mean ± SD for IFN-γ and TNF-α are shown for all six cell lines, background levels were subtracted using non-AML DAUDI cell line. Wilcoxon signed-rank test showed statistically significant difference in cytokines and chemokines produced in the culture supernatants of CD123 BATs plus AML cells or CD33GO BAT plus AML cells compared to the culture supernatants of unarmed ATC (UA) plus AML cells for IFN-γ and TNF-α for most cell lines (p value ranged from p<0.05 - p<0.00005). **(B)** Shows the chemokines MIP1-b and RANTES (pg/ml), background levels were subtracted using non-AML DAUDI cell line. Statistical analysis was performed using Wilcoxon signed-rank test. Chemokine MIP1-b was significantly higher (p value ranged from p<0.05 - p<0.0005) in the culture supernatants of CD123 BATs+AML cells and CD33GO BATs+AML cells compared to unarmed ATC (UA)+AML cells for NoMo1, EOL1 and KG1; RANTES showed significantly higher levels only in the culture supernatants of CD123 BATs+KG1 cells and CD33GO BATs+KG1 cells. **(C)** Shows the levels of immune suppressive sPD-L1 and IL-10 in the culture supernatants of CD123 BATs+AML cells, CD33GO BATs+AML cells and unarmed ATC (UA)+AML cells for all 6 cell lines. (* = p<0.05; ** = p<0.005; *** = p<0.0005; **** = p<0.00005)

To examine the effector activity of CD33GO BATs or CD123 BATs, 45 panels of cytokines and chemokines were measured after the *in vitro* 18-h cytotoxicity assay against TF1, NoMo1, EOL1, KG1, HL60, HL60/VCR, and an irrelevant cell line DAUDI. A large number of cytokines and chemokines were produced by CD33GO BATs or CD123 BATs when co-cultured with AML targets. Th_1_ effector cytokines interferon-γ (IFN-γ), tumor necrosis factor-α (TNF-α), and T-cell recruiting and activating chemokines macrophage inflammatory protein-1β (MIP-1β) and RANTES were the dominant cytokines and chemokines released by CD33GO BATs or CD123 BATs in response to AML cells in the culture supernatant (**Figures 3A, B**). All values were subtracted from the baseline control effector cells alone and target cells alone. The levels of IFN-γ and TNF-α were significantly higher in the culture supernatants of CD33GO BATs and NoMo1 (p < 0.0001; p < 0.0001), EOL1 (p < 0.02; p < 0.01), KG1 (p = 0.0002; p = 0.0003), HL60 (p = 0.0004; p = 0.003), and HL60/VCR (p < 0.005; p < 0.02) cells compared to the culture supernatant of unarmed ATCs and AML targets. Similarly, significantly higher levels of IFN-γ and TNF-α were present in the supernatants of CD123 BATs and TF1 (p < 0.01; p < 0.04), NoMo1 (p < 0.00001; p < 0.00001), EOL1 (p = 0.0001; p = 0.0002), KG1 (p < 0.0001; p < 0.0001), HL60 (p = 0.0004; p = 0.009), and HL60/VCR (p < 0.005; p < 0.03) cells compared to unarmed ATCs and AML targets (**Figure 3A**).

The levels of chemokine MIP-1β were significantly higher in co-cultures with CD33GO BATs and AML targets KG1 and NoMo1 or CD123 BATs and AML targets EOL1, KG1, and NoMo1 (p-value ranging from p < 0.05 to p < 0.0005) compared to unarmed ATCs and AML targets (**Figure 3B**). Co-culture of CD33GO BATs and DAUDI or CD123 BATs and DAUDI did not induce any cytokines or chemokines higher than the unarmed ATCs (data not shown).

In addition to Th_1_ cytokines and chemokines, the levels of Th_2_ cytokine IL-10 and checkpoint inhibitor soluble PD-L1 (sPD-L1) are shown in **Figure 3C**. The highest levels of both immune suppressive mediators were present in the culture supernatant of CD33GO BATs or CD123 BATs with NoMo1 cells. Culture supernatants from co-cultures of UAs with all cell lines also showed high levels of IL-10 and sPD-L1 (**Figure 3C**).

### Expression of CD33 and CD123 in primary acute myeloid leukemia samples

**Figure 4:**
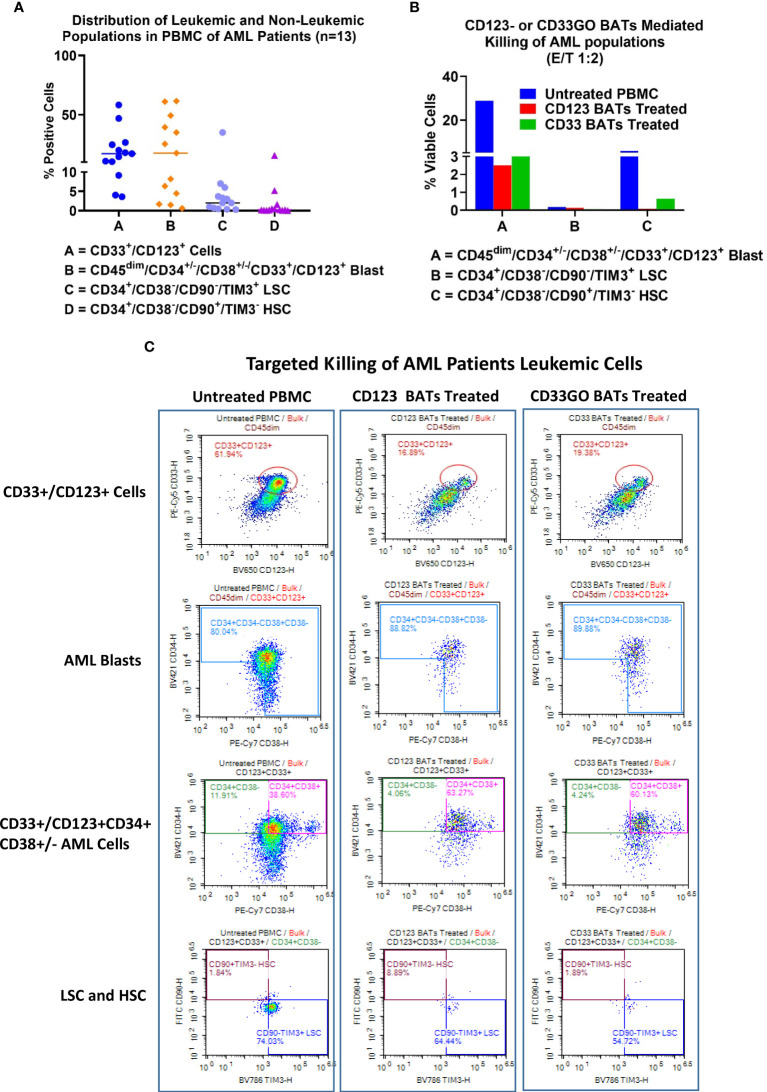
Cytotoxicity of AML patient derived PBMC. Cryopreserved PBMC from 13 AML patients from UVA were used as targets and CD33GO- or CD123 BATs as effectors at 1:1 E:T ratio for flow-based cytotoxicity assay. **(A)** Proportions of Blast and LSC in patient derived samples. Data show the proportions of CD33 and CD123, CD34+/CD38-, CD34+/CD33+ (Blasts), LSC and HSC in the primary AMLs samples. **(B)** Shows the targeted killing of AML blasts, AML leukemic stem cells and hematopoietic stem cells by CD33GO- and CD123 BATs compared to untreated PBMC from a patient. **(C)** Shows an examples of flow cytometry analysis of untreated (left panel), CD123 BATs (middle panel) and CD33GO BATs treated (right Panel) peripheral blood mononuclear cells (PBMC) sample (unique ID# 8500). Lower two plots of each panel show percentage of blasts, CD33+/CD123+ cells, HSC and LSC populations in untreated and BATs treated PBMC.

AML samples containing leukemic blasts and LSCs showed highly variable expression of CD33 and CD123. Expression of CD33^+^CD123^−^ and CD123^+^CD33^−^ cells ranged from 0.25% to 18.9% and 0.26% to 14.9%, respectively, and co-expression of CD33 and CD123 ranged from 3.6% to 58.2%. CD33- and CD123-expressing leukemic and non-leukemic populations ranged from 0.6% to 61.5% for blasts, 0.3% to 35% for LSCs, and 0.04% to 15.6% for HSC in the PBMCs from all 13 patients (**Figure 4A**).

### Expression and targeting of CD33- and CD123-positive blasts and leukemic stem cells

Examples of flow cytometry-based targeted killing of leukemic blasts and LSCs by CD123 BATs (middle panel) or CD33GO BATs (right panel) compared to untreated PBMCs (left panel) from one patient are shown in **Figure 4C**, **S2**. In each panel, four plots show gates for CD33^+^/CD123^+^ cells, blasts (CD45^dim^/CD33^+^/CD123^+^/CD34^+^/CD38^+/−^), LSCs (CD34^+^/CD38^−^/CD90^−^/TIM3^+^), and HSC (CD34^+^/CD38^−^/CD90^+^/TIM3^−^) populations. Targeted killing by CD123 BATs (middle panel) or CD33GO BATs (right panel) showed a drastic reduction in leukemic blasts and LSC populations compared to untreated control PBMCs (left panel) from the same patient; however, the HSC population for this patient was almost nil (**Figure 4C**). In **Figure 4B**, flow cytometry data of targeted killing of leukemic blasts, LSCs, and HSC of one patient by CD123 BATs or CD33GO BATs (**Figure 4C**) are displayed as a bar plot.

### Elimination of acute myeloid leukemia patients’ blasts and leukemic stem cells by CD33GO and CD123 bispecific antibody armed activated T cells

**Figure 5:**
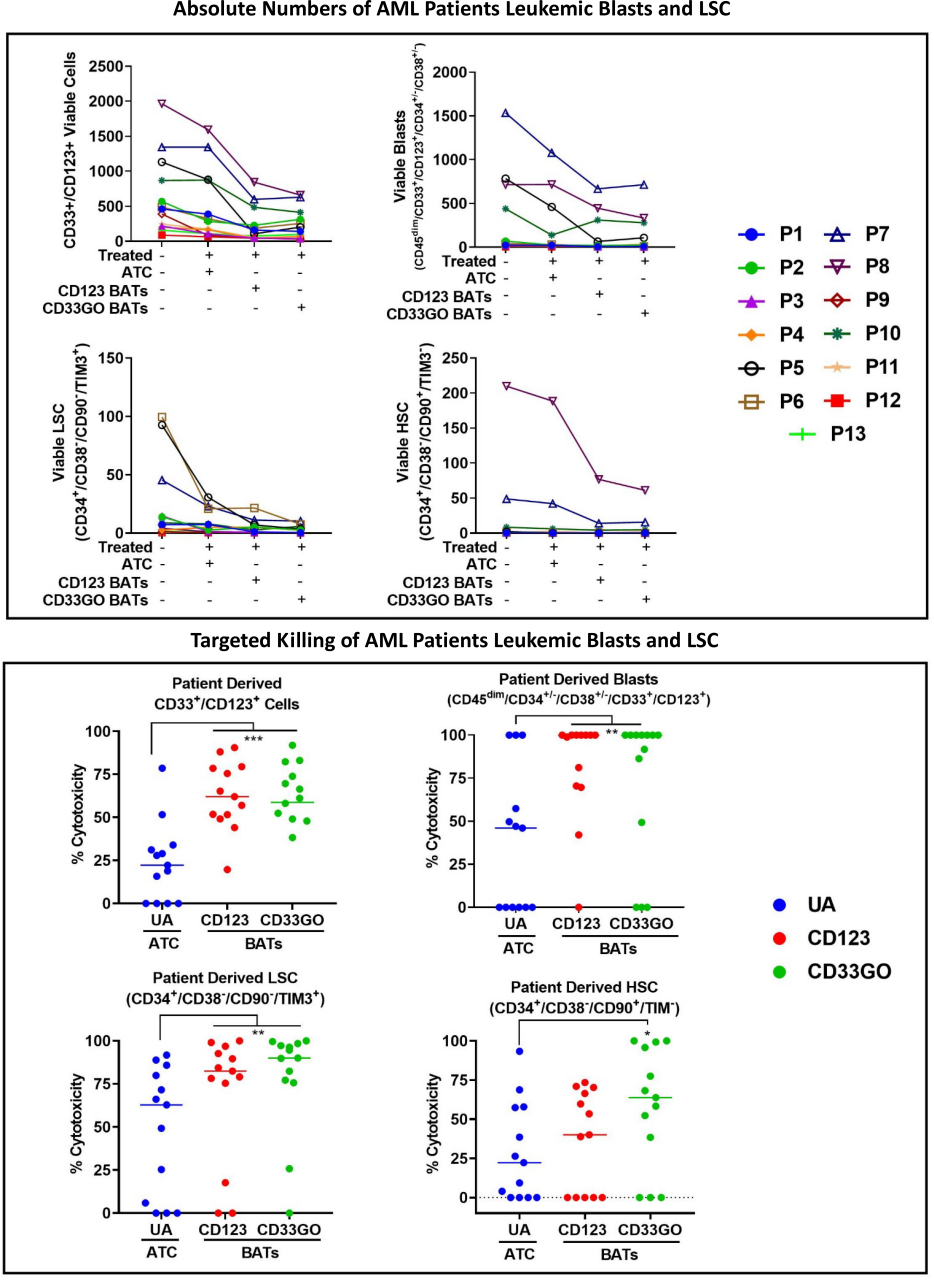
Cytotoxicity against Blasts and LSC by CD33GO and CD123 BATs. Upper panel shows the absolute numbers of remaining CD33+/CD123+ cells, leukemia precursors CD34+/CD38-, CD33+/CD34+/CD38- leukemic blast population, CD33+/CD123+/ CD34+/CD38-/CD90-/TIM3+ LSC and CD33+/CD123+/CD34+/ CD38-/CD90+/TIM3- HSC populations after targeted killing by ATC, CD33GO BATs or CD123 BATs compared to untreated PBMC. Lower panel shows the cytotoxicity against CD33/CD123 expressing cells by CD33GO- (p=0.0007) and CD123 BATs (p=0.0002); cytotoxicity against blast population by CD33GO- (p<0.005) and CD123 BATs (p<0.002); cytotoxicity against LSC by CD33GO- (p<0.006) and CD123 BATs (p<0.002) and cytotoxicity against HSC by CD33GO BATs (p<0.05) compared to unarmed ATC in the in vitro killing assay using Wilcoxon signed-rank test (* = p<0.05; ** = p<0.005; *** = p<0.0005).

In a cohort of 13 AML patients, there were variable levels of CD33 and CD123 expression (**Figure 4A**). PBMCs from AML patients were labeled with eFluor 450 tracking dye before overnight co-culture with BAT followed by staining for the remaining CD33^+^/CD123^+^ target cells expressing, leukemic blast, LSCs, and HSC populations in the co-culture compared to untreated PBMCs. The results are expressed as absolute numbers (**Figure 5**, top panel) and percent cytotoxicity of blasts, LSCs, and HSC (**Figure 5**, bottom panel) that are eliminated by BATs in a fixed volume of PBMCs using the flow cytometry-based cytotoxicity assay. Both CD33GO BATS and CD123 BATs showed significantly high cytotoxicity against CD33/CD123-expressing cells by CD33GO (p = 0.0007) and CD123 BATs (p = 0.0002) than unarmed ATCs. Likewise, significantly high cytotoxicity was observed against the blast population by CD33GO (p < 0.005) and CD123 BATs (p < 0.002) and against LSCs by CD33GO (p < 0.006) and CD123 BATs (p < 0.004) compared to unarmed ATCs in the *in vitro* killing assay of primary AML PBMCs specimens. However, it is important to mention that high cytotoxicity of AML cells was also exhibited by unarmed ATCs, suggesting that AML cells are sensitive to killing by LAK T cells. Since HSC population also expresses CD33 and CD123, targeting with CD33GO BATs showed significantly high cytotoxicity (p < 0.05) against HSC compared to unarmed ATCs; however, there was no significant difference in cytotoxicity observed between CD123 BATs and unarmed ATCs (**Figure 5**, bottom panel).

### HL60/VCR cells primed with bispecific antibody armed activated T cells retain drug

Next, we determined whether priming the HL60/VCR cells with CD33GO BATs or CD123 BATs can reduce the transporter pump activity to retain the fluorescent dye. Intriguingly, BAT-primed drug-resistant HL60/VCR cells showed increased fluorescence judged by MFI compared to untreated HL60/VCR cells (red bars; **Figure S3**). These data suggest that priming of drug-resistant cells with BATs can sensitize resistant AML cells for enhanced chemo-responsiveness. In addition, regardless of the high expression of drug efflux transporters, these drug-resistant cells can be efficiently targeted and killed by BATs.

### *In vivo* targeting of acute myeloid leukemia in NSG mice

**Figure 6:**
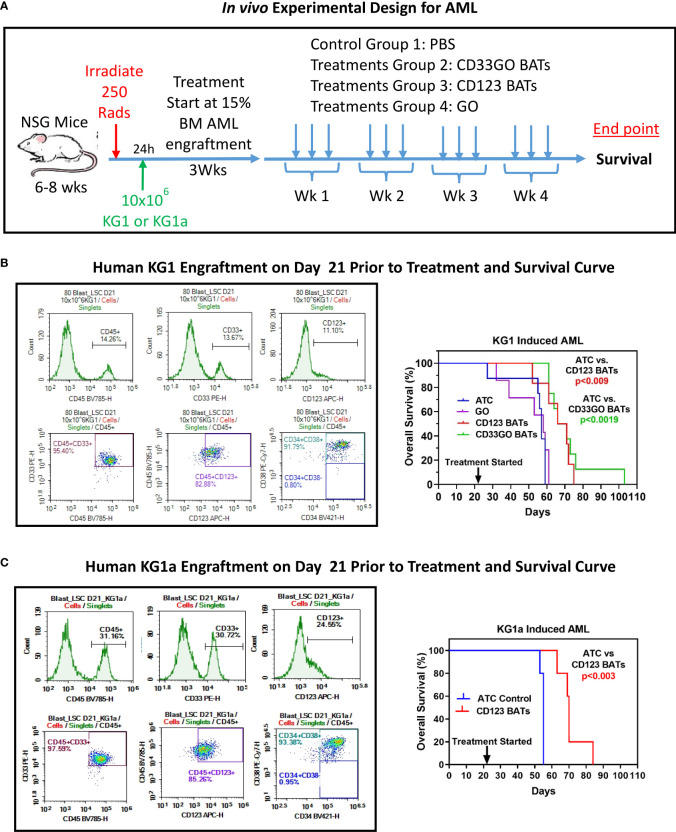
*In vivo* Efficacy of CD33GO and CD123 BATs against AML in NSG Mice. **(A)** Upper panel shows treatment schema. **(B)** NOD scid gamma ([NSG] NOD.Cg-Prkdcscid Il2rgtm1Wjl/SzJ) mice 6-8-week-old were irradiated with 250 Rads, after 24h of irradiation mice were intravenously injected with 10x106 KG1 cells to establish human AML graft (left panel). Right panel shows the percentage of overall survival of all four groups (n=10 mice/group). Mice treated with ATC or BATs received 20x106 cells/injection, 3x/week for 4 weeks and mice treated with GO (Mylotarg) received 0.06 mg/kg GO alone 3x/week for 4 weeks. Comparison of survival curve using Log-rank (Mantel-Cox) test show significantly prolonged survival of mice treated with CD33GO BATs (p<0.0001) compared to control ATC; CD123 BATs (p<0.0089) vs control ATC; CD33GO BATs (p<0.0006) vs. GO alone and CD123 BATs (p<0.01) vs. GO alone. **(C)** The results of in vivo experiment were confirmed using AML engraftment with KG1a cells in NSG mice (n=5). Mice were treated with control ATC or CD123 BATs on day 21 when almost 30% of CD45+ KG1a cells were engrafted in the bone marrow (left panel). Mice treated with ATC or BATs received 20x106 cells/injection, 3x/week for 4 weeks. Comparison of survival curve using Log-rank (Mantel-Cox) test show significantly prolonged survival of mice treated with CD123 BATs (p<0.003) compared to control ATC (right panel).

The experimental schema using NSG mice is shown in **Figure 6** (top panel) with the end point of the study to monitor the survival proportions in treated and control groups (n = 10 mice/group). Before starting the treatment with BATs, AML engraftment in mice was monitored by staining for human CD45^+^/CD33^+^/CD123^+^/CD34^+^/CD38^+^ cells in the flushed BM on days 7, 14, and 21 (**Figures 6**, **S4**). Engraftment of human CD45^+^ KG1 cells on days 7, 14 and 21 was <2%, 3.7%, and 14.2%, respectively. Treatment with 20 × 10^6^ cells/injection, 3×/week for 4 weeks, was started once engraftment of human CD45^+^ KG1 cells reached ~14%–15% (**Figure 6**, middle left panel). Control and treatment groups (G) include (G1) control, (G2) CD123 BATs, (G3) CD33GO BATs, and (G4) 0.06 mg/kg of Mylotarg (GO) alone. Following 4 weeks of treatment, mice were monitored for survival. All mice in control groups and ATC- and GO-treated mice died by day 40, while mice treated with CD123 BATs or CD33GO BATs survived up to 55+ and 80+ days, respectively, after treatment started on day 21 (**Figure 6**, middle right panel). Comparison of survival curve using a log-rank (Mantel–Cox) test showed a highly significant difference between mice treated with control ATCs and mice treated with CD33GO BATs (p < 0.0001), control ATCs *vs.* CD123 BATs (p < 0.0089), GO alone *vs.* CD33GO BATs (p < 0.0006), and GO alone *vs.* CD123 BATs (p < 0.01).

We confirmed the results of *in vivo* experiment again in the NSG mouse model using AML engraftment with KG1a cells instead of KG1 used in the above experiment. KG1a cell line is a well-characterized cell line for AML engraftment in xenograft mouse models (29). Because this cell line has a high expression of CD123, treatment was done with CD123 BATs and control ATCs. Mice were treated with control ATCs or CD123 BATs on day 21 when engraftment of CD45^+^ KG1a cells reached 30% (**Figure 6**, lower left panel) in the bone marrow. Data show significantly prolonged survival of mice treated with CD123 BATs (p < 0.003) compared to control ATCs (**Figure 6**, lower right panel).

## Discussion

There is an urgent need to develop new therapeutic agents to treat patients with AML. Despite over four decades of clinical research efforts, therapeutic options for AML remain limited. Chemotherapy for AML has not significantly improved outcomes (30–33), and the options for long-term cure in those who relapse after primary therapy are myeloablative chemotherapy and/or total body irradiation followed by AlloSCT in those who have an AlloSCT donor (2–5). Relapse arises out of minimal residual disease that is resistant to chemotherapy. However, for those who do not have an HLA-matched sibling or matched unrelated donors or who have significant organ dysfunction or are elderly, there are a few therapeutic options for this population (6, 8).

CD33 and CD123 are targets that are highly expressed in AML cells, and more than 80% of patients have AML cells that express both antigens (9–11). The addition of CD33GO or CD123 BATs after standard induction chemotherapy or after high dose chemotherapy followed by an autologous or allogeneic stem cell transplant may provide the additional anti-leukemic effect to eliminate minimal residual disease. Arming ATCs with CD33GOBi (CD33GO BATs) or CD123Bi (CD123 BATs) creates non-MHC restricted AML-specific cytotoxic T lymphocyte (CTL). In AML patient samples, CD33GO and CD123 BATs exhibit high levels of cytotoxicity directed at leukemic blasts and LSCs. Elimination of AML blasts and LSCs by CD33GO BATs and CD123 BATs was significantly higher compared to UAs.

CD33GO BATs and CD123 BATs mediated cytotoxicity was also evaluated against six AML cell lines; all six cell lines showed high levels of cytotoxicity except the TF1 cell line. Two of the cell lines HL60 and HL60/VCR showed very low expression of CD123 but showed high levels of effective specific cytotoxicity (**Table 1**). These results are consistent with our previous studies using HER2 BATs in HER2 (3+)-positive and HER2 (0–2+)-negative breast cancer patients in phase I clinical trial (21). In our preclinical study, EGFR BATs showed high levels of cytotoxicity against MCF-7 cells, which have a very low expression of EGFR and very low binding of cetuximab and rEGFR bispecific antibody (rEGFRBi). In spite of very low EGFR expression on MCF-7 cells, EGFR BATs showed high levels of specific cytotoxicity compared to non-specific cytotoxicity by unarmed activated T cells (Huang et al., 2022). Another study by Zitron et al. also showed high specific cytotoxicity by ATCs armed with HER2Bi and EGFRBi against primary glioblastoma cells in culture with low expression of both HER2 and EGFR (34). These studies support our data that show high levels of specific cytotoxicity by CD123 BATs against very low CD123-expressing cell lines (HL60 and HL60/VCR). It is clear that only a few molecules of target antigens are sufficient to trigger specific cytotoxicity, which may not be detectable by flow cytometry or immunohistochemistry.

*In vivo* study in NSG mice xenografted with AML showed significantly prolonged survival after multiple infusions of CD123 or CD33GO BATs. These *in vivo* data together with the *in vitro* data provide a strong rationale for testing BAT therapy in high-risk or refractory AML patients in combination with induction chemotherapy with autologous or allogeneic stem cell transplant rescue or treatment of patients who relapse after allogeneic stem cell transplant. Since allogeneic ATCs have been shown to suppress mixed lymphocyte culture responses, the use of allogeneic ATCs may provide an anti-leukemia effect and enhance hematopoietic and lymphopoietic recovery without causing graft-versus-host disease (GVHD) in the context of allogeneic stem cell transplant. This is supported by our earlier studies (35) that ATCs and armed ATCs were able to suppress allogeneic responses in mixed lymphocyte reaction (MLR) in six unrelated responder and stimulation combinations tested to determine whether random or non-HLA-matched normal donor samples could respond to host allogeneic (allo) antigens in patients undergoing allo-stem cell transplant. A possible explanation for the lack of response in our systems is that the precursor frequency of non-specifically expanded alloreactive T-cell clones is being suppressed by regulatory cells generated in the ATC culture (35).

We have shown previously that “priming” of the tumor cells with BATs lowers the threshold for effective cytotoxic doses (36). Standard induction chemotherapy, consisting of anthracycline and cytarabine, induces complete remissions in the majority of AML patients, but multidrug resistance (both intrinsic and acquired) in many patients limits the benefit of AML therapies in relapsed and refractory disease (37). Multidrug resistance (MDR) is mediated by various mechanisms involving numerous proteins belonging to a larger family of the ATP-binding cassette (ABC) transporter superfamily that plays a key role in drug efflux and multidrug resistance (38). Since the expression levels of MDR1 (ABCB1) and MRP1 (ABCC1) (39–41) transporter pumps regulate the anti-leukemic effects of GO by influencing the intracellular accumulation of calicheamicin, these transporter pumps play a critical role in drug resistance of AML. ABCB1 expression on blast cells varies from 19% to 75% and is expressed in more than 50% of AML patients (42, 43), which strongly correlates with response to GO. Here, we show that priming of GO and vincristine resistant cell line HL60/VCR with CD33GO BATs or CD123 BATs sensitizes GO resistant cells for enhanced chemo-responsiveness, suggesting that immunosensitization with BATs can result in a chemo-potentiation effect in drug refractory AML (**Figure S3**).

In our phase I trial, high risk/refractory NHL patients who received multiple infusions of ATCs armed with anti-CD3 × anti-CD20 bispecific antibody (CD20 BATs) given early after autologous SCT showed an anti-lymphoma effect (24). In a proof-of-concept phase Ib study, CD20 BATs infused twice prior to autologous stem cell transplant (auto-SCT) for high-risk MM patients showed that targeting of CD20^+^ clonogenic MM stem-like cells decreased the absolute number of clonogenic CD138^−^CD20^+^ cells in the bone marrow after two infusions and induced anti-MM immunity that could be transferred in the marrow graft and could be detected early and late after auto-SCT (25). Our phase I clinical trial in women with stage IV metastatic breast cancer (MBC) showed that multiple infusions of ATCs targeted with anti-CD3 × anti-HER2 BiAb induced the development of endogenous anti-tumor T-cell responses and induced a Th_1_ cytokine pattern (21, 26). In a subsequent proof-of-concept study involving five phase I MBC patients, “immune T cells” were obtained by the second apheresis after HER2 BAT infusions and expanded to produce immune ATCs that were infused after auto-SCT (22). Immune testing after SCT showed accelerated and enhanced reconstitution of specific anti-breast cancer cellular and humoral responses to different epitopes of cancer antigens. Together, preclinical and clinical data show that specific immunity was induced by multiple infusions of HER2 BATs and that the specific cellular immunity could be transferred by immune ATC infusions and B cells in the stem cell graft.

Anti-CD33- or anti-CD123-targeted biologics in AML using bispecific antibodies and CAR T cells show feasibility and safety but are yet to show efficacy (13–16). CRS and loss of graft function have been challenging dose-limiting factors for biologics. A recent phase I/II study using flotetuzumab (anti-CD3ϵ × anti-CD123) in 88 adults with relapsed/refractory AML with primary induction failure and relapse after <6 months showed an acceptable safety profile and encouraging evidence of clinical activity (15).

**Figure 7:**
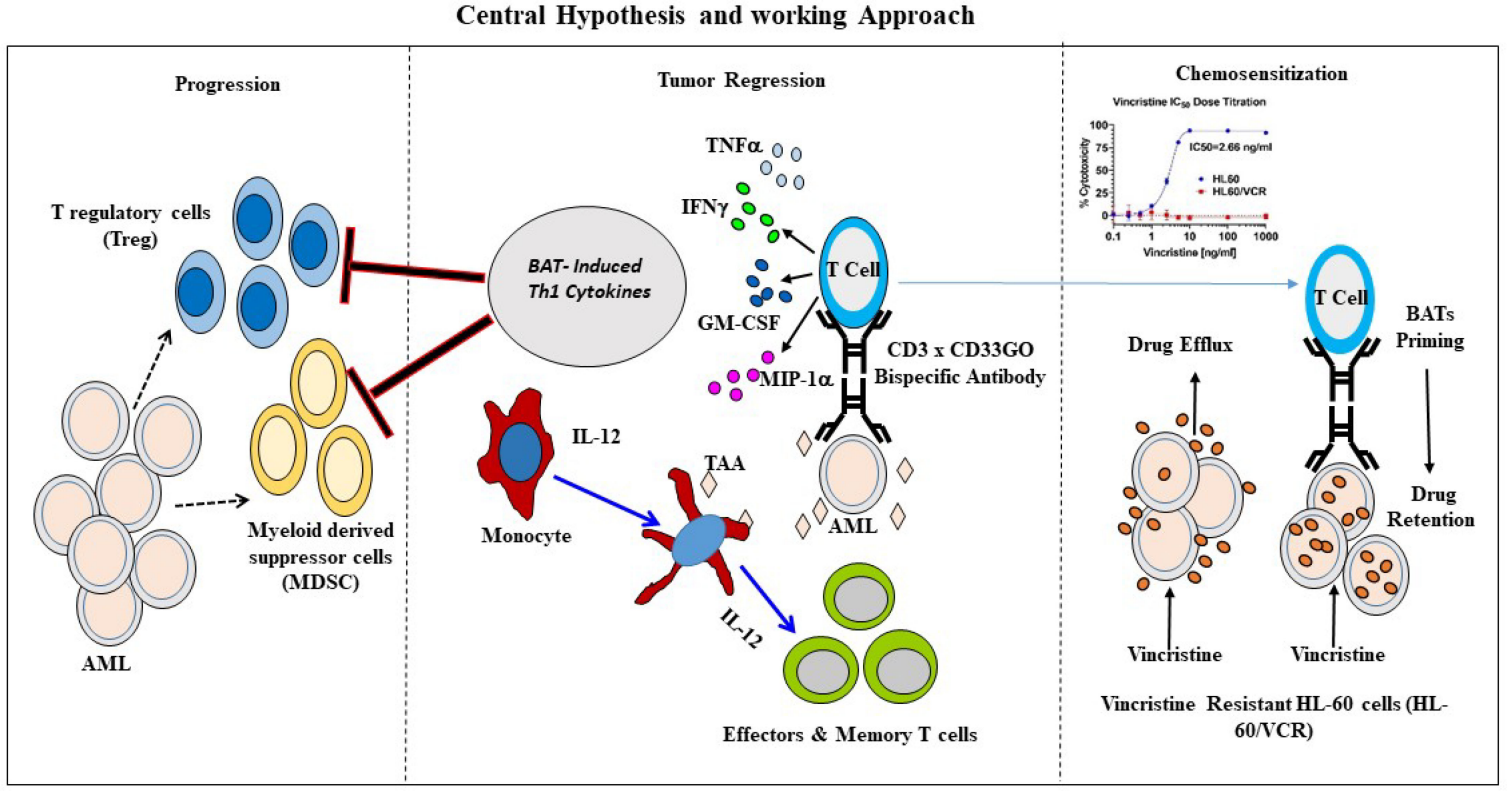
Schematic representation of BATs mediated antitumor effects. Middle panel shows an overview of immunotherapy approach to re-direct T cells using bispecific antibody. Arming of polyclonally activated T cells (ATC) with bispecific antibody combines the targeting specificity of mAbs and non-MHC-restricted cytotoxicity of T cells. BATs engagement to tumor cells kills target cells releasing tumor antigens as well as cytokines/chemokines in the TME. Chemokines recruits endogenous immune cells in the TME. Cytokines GM-CSF and TNF-a stimulates monocytes for activation and maturation into antigen presenting cells (APC) with eventual antigen presentation by APC to naïve T cells, which leads to the clonal expansion of tumor-specific effectors and central memory cells differentiation, and development of long-term anti-tumor immunity. Left panel showing that Th1 cytokine enriched microenvironment inhibits immune suppressor cells such as T regulatory cells (Tregs) and myeloid derived suppressor cells (MDSC) thus enhancing anti-tumor activity by BATs. Right Panel shows that priming of the vincristine resistant AML cells (HL-60/VCR) with BATs can “sensitize” drug resistant cells for effective killing by chemotherapy drug by downregulating expression of MDR related proteins responsible for chemo drug efflux.

This proof-of-principle study shows that BAT-based therapeutic strategy, consisting of multiple non-toxic infusions, serial cytotoxicity limited by arming dose, and recruitment of the endogenous adaptive and innate immune cells to provide an anti-leukemia effect, may provide simple, inexpensive, and potent biologics for the treatment of AML. CD33GO or CD123 BATs could be used not only in combination with chemotherapy but also as immune consolidation after high-dose chemotherapy followed by allogeneic SCT to improve remission intervals and overall survival. More importantly, BAT therapy may sensitize cancer cells for enhanced chemo-responsiveness and may induce long-term anti-AML immunity. **Figure 7** shows the anti-AML effects of our approach.

## Data availability statement

The raw data supporting the conclusions of this article will be made available by the authors, without undue reservation.

## Ethics statement

This study was reviewed and approved by AML cells from pretreatment diagnostic peripheral blood or bone marrow specimens were obtained from Orion biorepository at University of Virginia Cancer Research Center from adult patients with AML. Patients provided written informed consent for the collection and use of their biospecimens for research purposes under protocols approved by the UVA Institutional Review Board. Clinical data were de-identified for all patients. The patients/participants provided their written informed consent to participate in this study. Animal protocol was reviewed and approved by the Institute of Animal Care and Use Committee of the University of Virginia.

## Author contributions

LL and AT conceived the idea. AT wrote the manuscript. AT, LL, MH, and EK designed the experiments and performed the data analysis. EK performed the experiments. All authors contributed to the article and approved the submitted version.

## Funding

This study was primarily supported by funding from in part by R01 CA92344, R01 CA140314, R01 CA182526, the UVA Cancer Center Support Grant P30 CA044579, and startup funds from the University of Virginia Cancer Center.

## Acknowledgments

The authors thank the UVA Oncology Research Information Exchange Network (ORIEN) Team and UVA Biorepository and Tissue Research Facility (BTRF) with Partners in Discovery for Total Cancer Care at UVA protocol IRB HSR 18445 for the consent, specimen procurement and processing, and access to the clinical samples.

## Conflict of interest

LL and MH are co-founders of TransTarget, Inc., and LL serves on the SAB for Rapa Therapeutics. AT is a co-founder of AlphaImmunePlatform LLC.

The remaining author declare that the research was conducted in the absence of any commercial or financial relationships that could be construed as a potential conflict of interest.

## Publisher’s note

All claims expressed in this article are solely those of the authors and do not necessarily represent those of their affiliated organizations, or those of the publisher, the editors and the reviewers. Any product that may be evaluated in this article, or claim that may be made by its manufacturer, is not guaranteed or endorsed by the publisher.
