# Supplementary material for: Bispecific antibody-targeted T-cell therapy for acute myeloid leukemia
Source: Front Immunol. 2022 Nov 1;13:899468. doi: 10.3389/fimmu.2022.899468 (PMC9663847; doi:10.3389/fimmu.2022.899468)

Methods

IC_{50} Dose Determination for GO in AML Cell Lines. Cell lines were incubated with the range of concentration (0.01, 0.1, 1, 10, 100, 1000, 10,000 ng/mL) of GO for 72h followed by flow-based cytotoxicity assay. IC_{50} values were calculated by non-linear regression analysis of dose versus response of a logarithm-transformed data. IC_{50} values were calculated based on 50% cytotoxicity.

Flow Cytometry-Based Drug Efflux Assay. Cell-based multi drug resistance (MDR) direct dye efflux assay (ECM910, Millipore) was performed to determine whether BATs primed GO resistant HL60/VCR cell line (vincristine resistant HL60 cells) can become chemo-responsive as described[34]. In this assay, the efflux activity of ABC transporters is optimal at 37ºC and ineffective at 4ºC. HL60/VCR and HL60 cells are preloaded with MDR1 fluorescent substrates retain dye and exhibit high fluorescence when incubated at 4ºC while cells incubated at 37 ºC will efflux the dye and show reduced fluorescence. The persistent intracellular accumulation of Rhodamine 123 and $DiOC_2$ in HL60/VCR and HL60 shows resistance by rapid efflux whereas delayed efflux shows impaired MDR pump function at 37ºC. Flow data was acquired by NovoCyte flow cytometer and analyzed using NovoExpress software.

Cytokines/Chemokines Profile in the Supernatant of Target and Effector Cells Co-cultures. CD33GO BATs, CD123 BATs, or unarmed ATC were co-cultured overnight in the presence of AML targets and the cell-free supernatants were quantitated for 45-panel (R&D Systems) cytokines, chemokines and growth factors using a Bio-Plex 200 system (BIO-RAD, Hercules, CA). The values are reported in pg/mL of culture supernatants, effector cells and target cells alone served as controls.

Table S1. Specific and common surface biomarkers to identify normal and leukemic cell populations.

Cell Type	Specific Phenotype Markers	Common Phenotype Markers
AML Blasts	$CD34^{+}/CD38^{-}$	CD33/CD123
AML Leukemic Stem cells (LSC)	$CD34^{+}CD38^{-}/CD90^{-}/TIM3^{+}$	CD33/CD123
Hematopoietic Stem cells (HSC)	$CD34^{+}CD38^{-}/CD90^{+}/TIM3^{-}$	CD33/CD123

Figure Legends

Figure S1. A) Shows the expression of CD33 and CD123 on KG1, EOL1, NoMo1, TF1, HL60 and HL60/VCR (*right dotplots*). Left plots show proportions CD34+/CD38-, CD34+/CD38+ and $CD34^{-}/CD38^{+}$ cells in all cell lines. **B)** Shows the bar plot of subpopulations gated on CD33, CD123, CD34 and CD38 phenotype in all six cell lines.

Figure S2. Drug Efflux Assay. Vincristine and GO resistant HL-60/VCR AML cell lines primed with BATs show high cellular uptake and retention of Rhodamine 123 compared to control unprimed HL-60/VCR cells. High fluorescence (elevated MFI) after priming with BATs suggest decreased activities of efflux transporters, MRD1 and MRP1 pumps.

Figure S3. Engraftment of AML cells NSG Mice. NOD *scid* gamma ([NSG] NOD.Cg-$Prkdc^{scid}$ $Il2rg^{tm1Wjl}$/SzJ) mice 6-8-week-old were irradiated with 250 Rads, after 24h of irradiation mice were intravenously injected with $10x10^{6}$ KG1 cells to establish human AML graft. The AML engraftment was monitored by staining for human $CD45^{+}$ cells in the flushed bone marrow (BM) on days 7, 14, and 21, treatment was started once ~15% human $CD45^{+}$ cells were engrafted. Flow cytometry plots show engraftment of human $CD33^{+}$ $CD123^{+}$ $CD45^{+}$ cells on day 21.

Figure S1

A. Co-Expression of CD33 and CD123 and Proportions of Blast in Cell lines

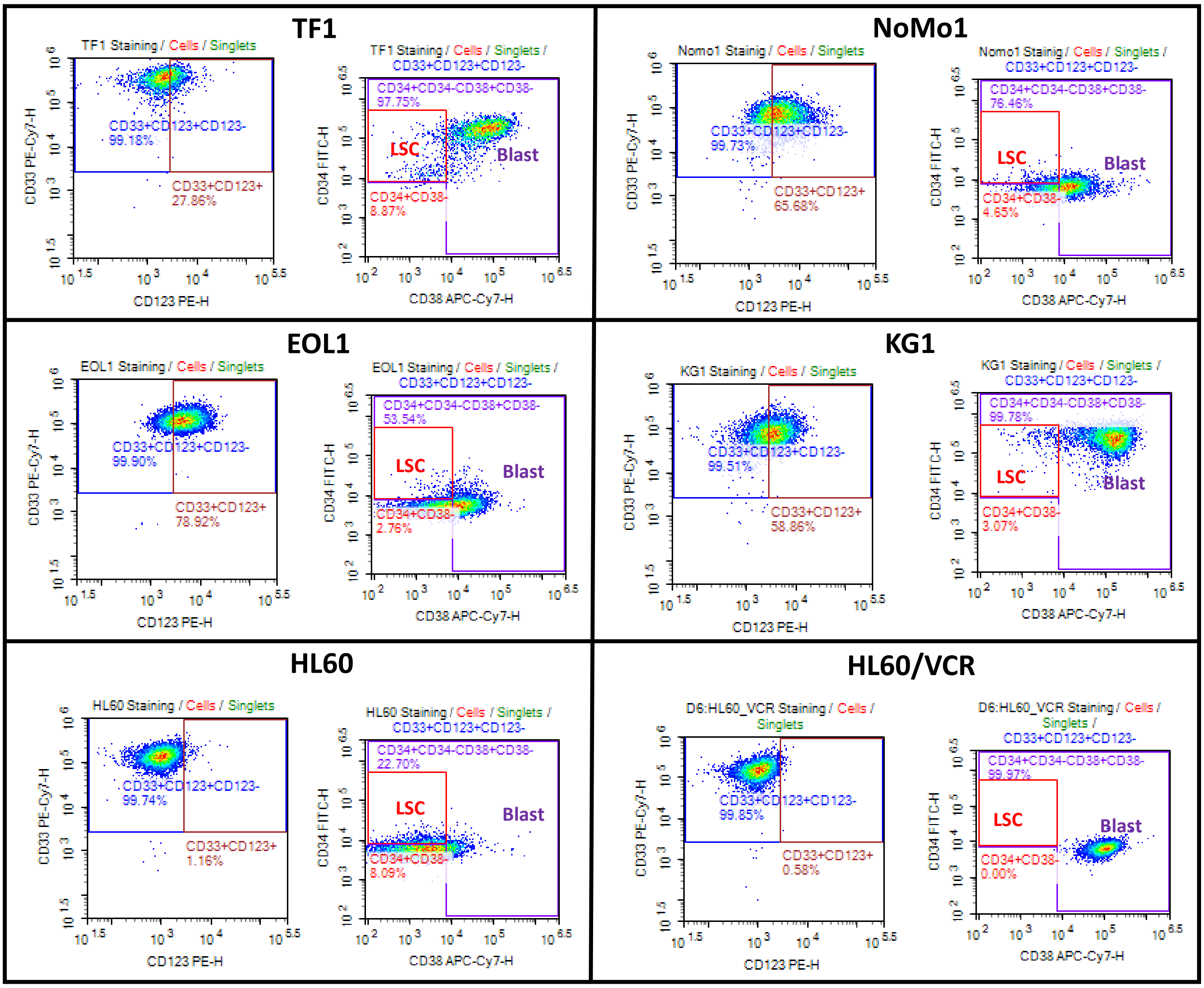

B. Phenotype of AML Cell Lines

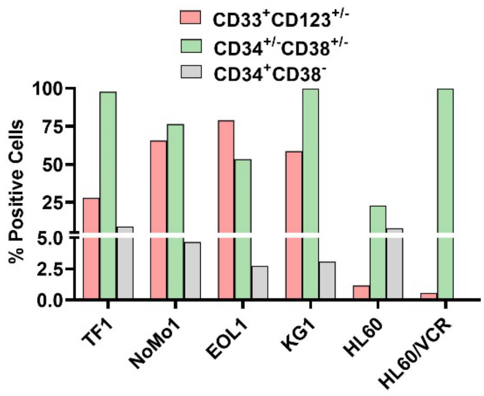

Figure S2

Targeted Killing of AML Patients Leukemic Cells

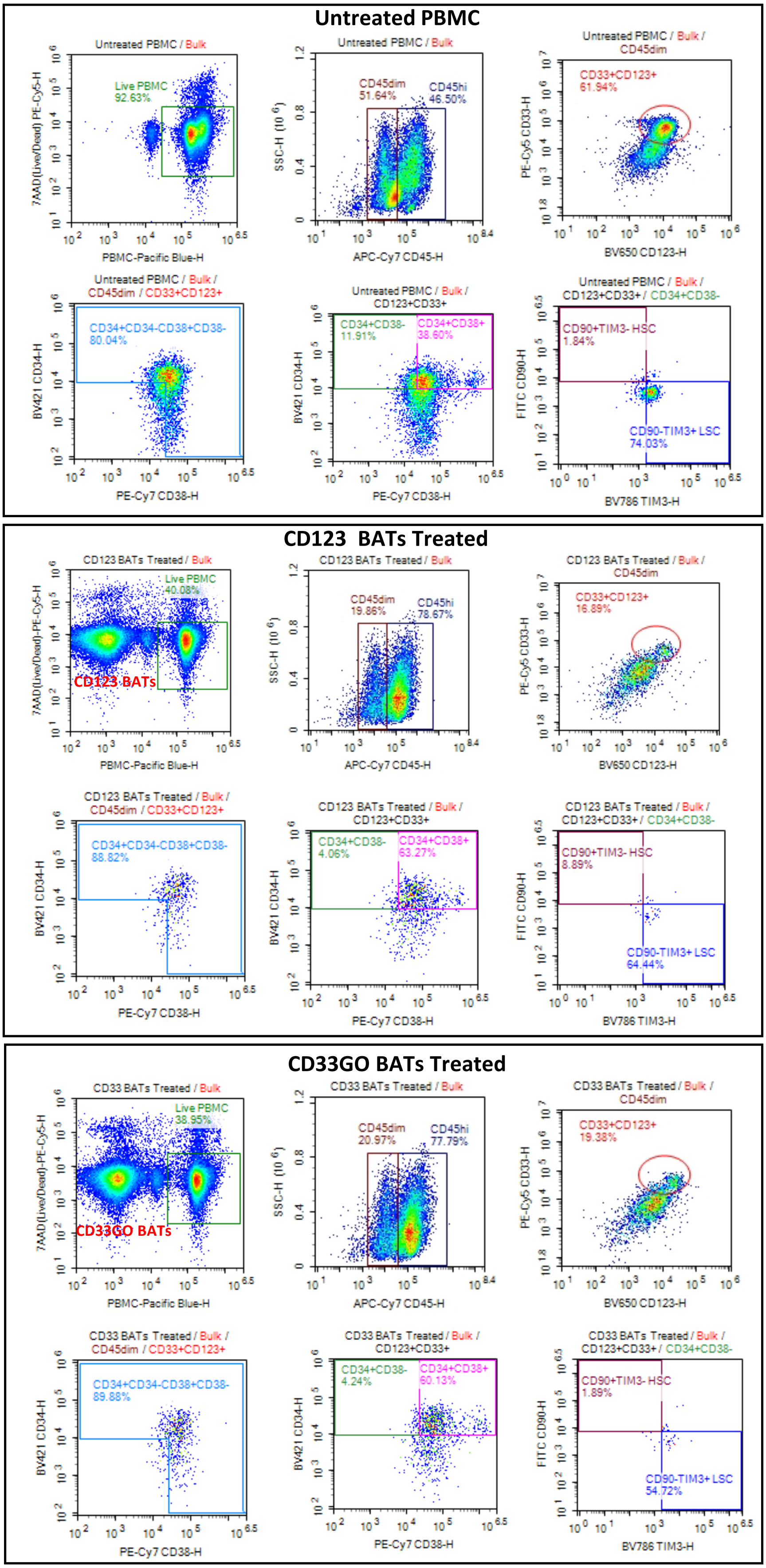

Figure S3

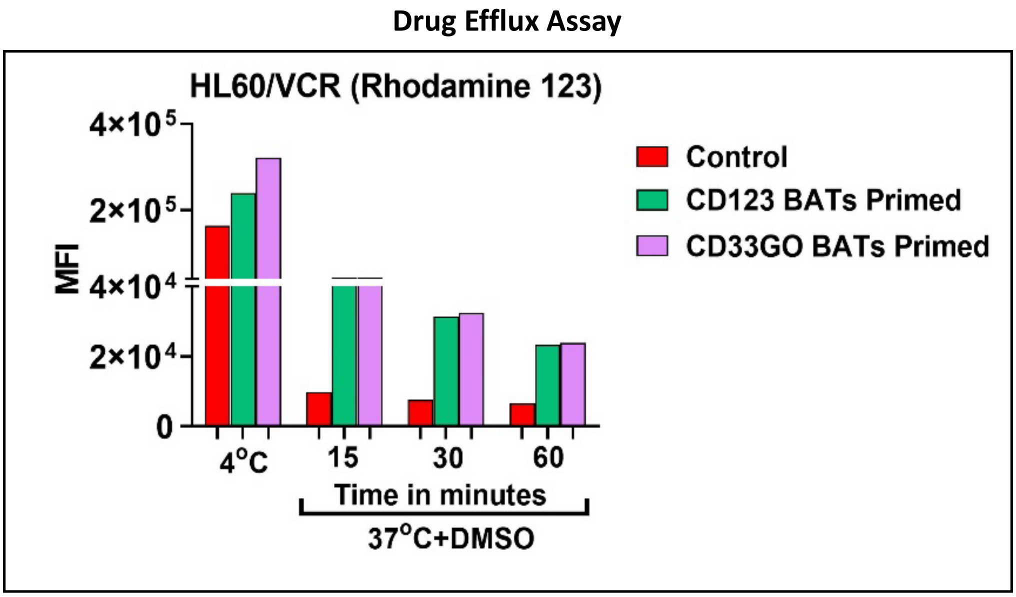
Drug Efflux Assay
HL60/VCR (Rhodamine 123)
4×10^5
2×10^5
4×10^4
2×10^4
0
MFI
Control
CD123 BATs Primed
CD33GO BATs Primed
4°C
15
30
60
Time in minutes
37°C+DMSO

Figure S4

Engraftment on Day 7

Engraftment on Day 14

Engraftment on Day 21

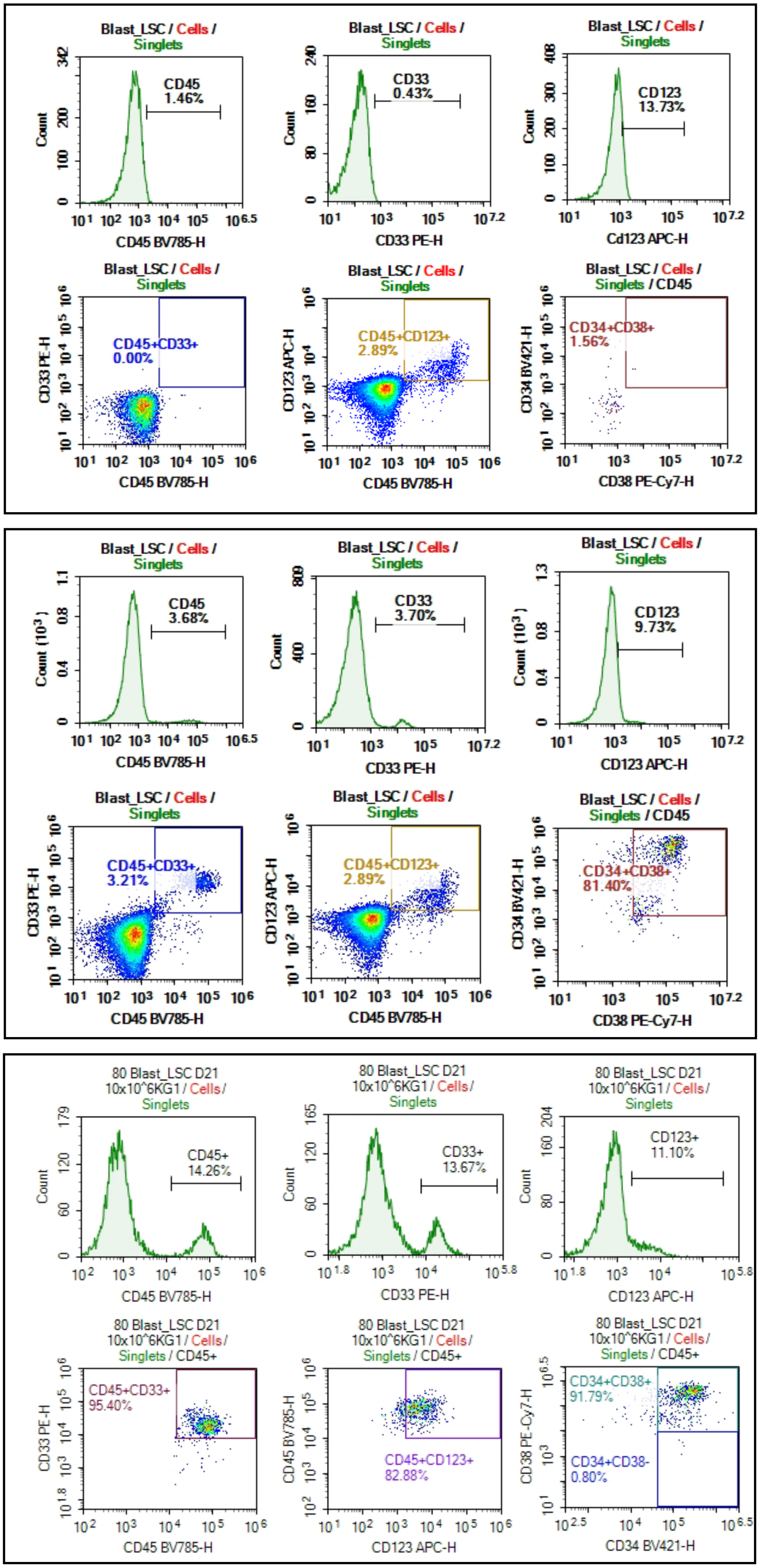

Supplement: Supplementary file 1 [file DataSheet_1.pdf]
